# Supplementary material for: Molecular Docking and Dynamics Simulation of Natural Compounds from Betel Leaves (Piper betle L.) for Investigating the Potential Inhibition of Alpha-Amylase and Alpha-Glucosidase of Type 2 Diabetes
Source: Molecules. 2022 Jul 15;27(14):4526. doi: 10.3390/molecules27144526 (PMC9316265; doi:10.3390/molecules27144526)
Supplement: Supplementary file 1 [file molecules-27-04526-s001.zip › molecules-1759773-supplementary.pdf]

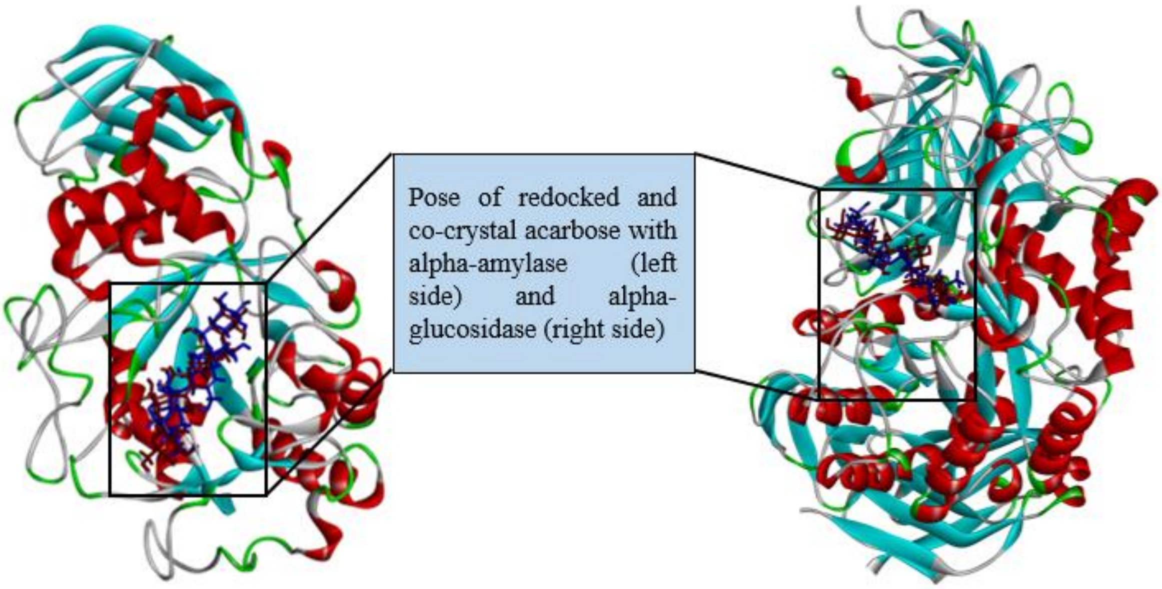

Figure S1. Validation of docking protocol using redocking. (A) Green colour denotes the x-ray co-crystal acarbose and dark red denotes after redocking with alpha amylase. The RMSD difference was 2.25 Å. (B) Green colour denotes the x-ray co-crystal acarbose and dark red denotes after redocking with alpha glucosidase. The RMSD difference was 2.6 Å.

Table S1. List of physicochemical, pharmacokinetics, and other properties of the promising drug compound (Apigenin-7-O-glucoside) and control acarbose

**Parameters**	**Characteristics**	**Apigenin-7-O-glucoside (best compound)**	**Acarbose (control compound)**
Physicochemical Properties	Formula	C21H20O10	C25H43NO18
	Molecular weight	432.38 g/mol	645.60 g/mol
	Num. heavy atoms	31	44
	Num. atom. heavy atoms	16	0
	Fraction Csp3	0.29	0.92
	Num. rotatable bonds	4	9
	Num. H-bond acceptors	10	19
	Num. H-bond donors	6	14
	Molar refractivity	106.11	136.69
	TPSA	170.05 $Å^2$	321.17 $Å^2$
Lipophilicity	Log Po/w (iLOGP)	2.17	0.63
	Log Po/w (XLOGP3)	1.81	-8.53
	Log Po/w (WLOGP)	0.05	-8.56
	Log Po/w (MLOGP)	-1.61	-6.94
	Log Po/w (SILICOS-IT)	0.35	-7.69
	Consensus Log Po/w	0.55	-6.22
Water Solubility	Log S ESOL	-3.78	2.13
	Solubility	7.19e-02 mg/ml; 1.66e-04 mol/l	8.61e+04 mg/ml; 1.33e+02 mol/l
	Class	Soluble	Highly soluble
Pharmacokinetics properties	GI absorption	37.609(%)	Low
	BBB permeant	No	No
	Pgp substrate	Yes	Yes
	CYP1A2 inhibitor	No	No
	CYP2C19 inhibitor	No	No
	CYP2C9 inhibitor	No	No
	CYP2D6 inhibitor	No	No
Drug likeliness activity	Lipinski #violations	Yes; 1 violation: NH or OH>5	No; 3 violations: MW>500, N or O>10, NH or OH>5
	Veber #violations	Veber No; 1 violation: TPSA>140	No; 1 violation: TPSA>140
	Bioavailability score	Bioavailability Score 0.55	0.17
Toxicity	AMES toxicity	No	No
	Max. tolerated dose (human)	0.515 (log mg/kg/day)	0.435 (log mg/kg/day)
	hERG I inhibitor	No	No
	Hepatotoxicity	No	No
	Skin sensitization	No	No
